# Changes in Hippocampal Plasticity in Depression and Therapeutic Approaches Influencing These Changes

**DOI:** 10.1155/2020/8861903

**Published:** 2020-11-26

**Authors:** Wenbo Xu, Xiaoxiao Yao, Fangyi Zhao, Haisheng Zhao, Ziqian Cheng, Wei Yang, Ranji Cui, Songbai Xu, Bingjin Li

**Affiliations:** ^1^Jilin Provincial Key Laboratory on Molecular and Chemical Genetic, The Second Hospital of Jilin University, Changchun, China; ^2^Department of Neurosurgery, First Hospital of Jilin University, Changchun, China

## Abstract

Depression is a common neurological disease that seriously affects human health. There are many hypotheses about the pathogenesis of depression, and the most widely recognized and applied is the monoamine hypothesis. However, no hypothesis can fully explain the pathogenesis of depression. At present, the brain-derived neurotrophic factor (BDNF) and neurogenesis hypotheses have highlighted the important role of plasticity in depression. The plasticity of neurons and glial cells plays a vital role in the transmission and integration of signals in the central nervous system. Plasticity is the adaptive change in the nervous system in response to changes in external signals. The hippocampus is an important anatomical area associated with depression. Studies have shown that some antidepressants can treat depression by changing the plasticity of the hippocampus. Furthermore, caloric restriction has also been shown to affect antidepressant and hippocampal plasticity changes. In this review, we summarize the latest research, focusing on changes in the plasticity of hippocampal neurons and glial cells in depression and the role of BDNF in the changes in hippocampal plasticity in depression, as well as caloric restriction and mitochondrial plasticity. This review may contribute to the development of antidepressant drugs and elucidating the mechanism of depression.

## 1. Introduction

According to the latest epidemiological survey, the incidence of depression worldwide is 4.7%, and the incidence in China is 4%; depression is a serious disease, and only 10% of patients respond effectively to treatment [1]. Depression severely affects human health and quality of life, including but not limited to excessive negative emotions, anhedonia, and cognitive impairment. The hippocampus is a key anatomical brain region associated with depression. Numerous studies have confirmed that changes in hippocampal plasticity (hippocampal volume, number of synapses, synaptic plasticity, changes in glutamate receptors, neurogenesis, and glial cell plasticity) occur in patients with depression [2]. It has been reported that the volume of the hippocampus in patients with major depression disorder (MDD) is significantly reduced [3]. After using antidepressant drugs, patient hippocampal tail volume increases in proportion to symptom relief [4]. The change in volume could be due to changes in neurons and glial cells.

Studies have shown that stress (especially chronic stress and early life stress) is closely related to the development of depression [5]. Chronic stress can cause apoptosis in hippocampal subregions and affect the integrity of hippocampal cells [6]. Long-term potentiation (LTP) and long-term depression (LTD) are the two main forms of synaptic plasticity changes. Stress causes changes in synaptic efficacy and stimulates the hypothalamic-pituitary-adrenal (HPA) axis to increase the levels of glucocorticoid (cortisol in human, corticosterone in rodents), resulting in a decrease in hippocampal LTP [7] that contributes to LTD [8]. Cell-level studies have found that stress leads to decreases in dendritic complexity and dendritic spine density [9]. On the other hand, excitatory synaptic neurotransmission is also affected in chronically stressed animals [10]. Central nervous system neurotransmitter dysfunction is one of the aspects of the pathogenesis of depression. Glutamate is widely distributed in the central nervous system and is an important excitatory neurotransmitter. Glutamate works by binding to the corresponding glutamate receptors, and pharmacological studies have shown that glutamate receptor modulators have antidepressant effects.

There are many hypotheses about the pathogenesis of depression, but the biological mechanisms remain unclear. The monoamine hypothesis is the most widely accepted hypothesis, and most current treatments for depression are based on the monoamine hypothesis. However, there are many limitations to the monoamine hypothesis. For example, the latency of antidepressant drugs cannot be explained, not all drugs that enhance monoamine functions have antidepressant activity, and a reduction in monoamines does not induce depression in healthy humans [11]. The neuroendocrine hypothesis proposes that abnormal function of the HPA axis leads to depression. Patients with depression often exhibit excessive secretion of glucocorticoids [12]. In a rodent model of chronic stress (classic depression model), plasma corticosterone levels are significantly increased [13]. Long-term administration of exogenous corticosterone can cause depression-like behavior in rodents [14]. With the development of immunological research, clinical studies have shown that chronic inflammation and some viral infectious diseases can significantly increase the incidence of depression, and the neuroinflammatory response hypothesis has thus been proposed. Studies have shown that the levels of proinflammatory cytokines in patients with depression are significantly increased [15]. Proinflammatory cytokines can affect the metabolism of monoamine transmitters and can also act on the HPA axis, impairing the negative feedback regulation of the HPA axis [16].

In recent years, people have gradually realized that information processing in the brain is not just the transfer of chemicals between neurons, but the result of the complex effects of neural networks. This finding puts forward the concept of neuroplasticity, that is, the ability of the nervous system to adapt and respond to the environment, including neurogenesis, neuronal remodeling, and synapse formation. The theory proposes that neuroplasticity disorders are involved in the pathological process of depression and that neurotrophic factors are important indicators for evaluating neuroplasticity. Brain-derived neurotrophic factor (BDNF) is a kind of neurotrophic factor. BDNF plays a key role in regulating synapse, hippocampal LTP, and neurogenesis [17].

Unfortunately, there is no single hypothesis that can explain the pathogenesis of depression. Moreover, the current clinical antidepressant treatment method is simplistic, the cure rate of antidepressant drugs is low, their side effects are high, and they are costly. The role of calorie restriction (CR) in depression has recently been revealed [18]. Previous research confirmed that CR is positively correlated with lifespan and negatively correlated with neurological diseases [19]. Studies have shown that CR can increase hippocampal neurogenesis and BDNF expression [20]. Neurogenesis is closely related to depression. Mitochondria are the sites at which cells metabolize energy and control programmed cell death [21]. The mitochondrial network plays a key role in CR [22]. Therefore, in addition to exploring the effect of CR on hippocampal plasticity, we also discuss the role of mitochondrial plasticity of the hippocampus.

At present, there have been many studies on antidepressants, and there are also different views on the pathogenesis of depression. However, the shortcomings of antidepressants are obvious to everyone. Because CR is simple to implement and has almost no side effects, it may be used to treat depression or to assist with existing antidepressant treatments to improve their efficacy. In this review, we introduce the various changes in hippocampal plasticity in depression and discuss the role of BDNF and CR in the treatment of depression. This review of hippocampal neuroplasticity may contribute to the development of new drugs and treatments.

## 2. Changes in Hippocampal Plasticity in Depression

The hippocampus is part of the limbic system and plays a vital role in depression. Numerous changes in hippocampal plasticity can be seen in both human depression patients and rodent depression models. There is a significant reduction in hippocampal volume. Depression can cause changes in various subregions, glutamate receptors, and glial cells in the hippocampus. Stress exists at the core of the many influencing factors. Some antidepressants also act through plasticity regulation. The different structures and functions of different hippocampal subregions may help to better understand hippocampal plasticity.

### 2.1. Hippocampal Volume

Clinical studies and neuroimaging results have shown that the hippocampal volume of patients with depression is reduced [23]. Similar results were observed in rodent models [24]. Both unipolar and bipolar depression (BD) patients have decreased hippocampal volume [25]. Telomere length is positively correlated with hippocampal volume, and reduced telomere length increases the risk of BD [26]. Chronic stress reduces hippocampal volume and inhibits neurogenesis in rats [27]. Early life stress increases the risk of MDD, which is associated with reduced left hippocampal volume [28]. The relationship between hippocampal volume and depression seems very complicated. Electroconvulsive therapy (ECT) increases the volume of the left hippocampus in patients, but there is no positive correlation with the treatment effect on depression [29]. The reduction in the hippocampus is not only caused by depression but is also a cause of depression [30]. Moreover, changes in hippocampal volume in depression are regulated by gene polymorphisms and gene expressions such as oxytocin receptor genes [31], monoamine-related genes [32], and neuroinflammatory genes [33].

### 2.2. Hippocampal Synaptic Neuroplasticity in the Different Subregions

In depression, the changes in hippocampal synaptic plasticity are also reflected in the hippocampal subregions, especially the cornu ammonis 3 (CA3) and the dentate gyrus (DG). In this section, we summarize the relationship between different hippocampal subregions and LTP and LTD and introduce some antidepressant drugs or methods to improve depression (based on the plasticity of the different subregions).

#### 2.2.1. Cornu Ammonis (CA1-CA3)

CA1 neurons accept 2 different glutamatergic inputs: temporoammonic- (TA-) CA1 and the CA3 region and the Schaffer collateral- (SC-) CA1. The JAK-STAT signaling pathway plays a role in the LTD induced by both pathways [34]. Chronic unpredictable stress (CUS) and corticosterone administration can reduce the excitatory signaling of TA-CA1 in rats [35]. Long-term plasticity in CA1 is induced by GABAergic interneurons (parvalbumin-expressing (PV+), nitric oxide synthase-expressing (NOS+)) [36]. Acute stress activates *μ*-opioid receptors on GABAergic neurons to facilitate low-frequency stimulation-induced LTD at SC-CA1 glutamatergic synapses in mice [37]. Electroacupuncture (EA) can improve depression by restoring CA1 synaptic plasticity, which may be mediated by regulating 5-HT receptor levels [38]. The perineuronal net (PNN) restricts LTD in the CA1 area in mice [39]. Interlaminar CA1-CA1 networks exhibit NMDA receptor-dependent LTP rather than LTD [40].

CA2 is a region that tends to be overlooked. The volume of CA2 is negatively correlated with depressive symptoms [41]. It is difficult to induce LTP and LTD in the CA2 region, which showed unique synaptic stability compared with that of other regions. A study showed that neurons in CA2 require more current to generate action potentials [42]. The function of CA2 in regulating synaptic plasticity may be different from other CA regions. The inhibitory LTD of PV+ interneurons in the CA2 region does not exist in young mice and may require the maturation of PNN and ErbB4 [43]. The plasticity of PV+ neurons is mediated by *δ*-opioid receptors [44].

CA3 pyramidal cells receive three types of excitatory synaptic afferent neurons: mossy fibers (MFs), perforant paths (PPs), and associational/commissural (AC) fibers. The MF-CA3 synapse has been reported to play a role in antidepressant drugs [45]. Novel spatial learning contributes to hippocampal plasticity and is termed learning-facilitated plasticity (LFP). When rats explore different environments, the MF-CA3 and AC-CA3 synapses show different responses to LTD. However, when exploring a new empty environment, both synapses promote LTP [46]. LFP-induced LTD and LTP in the MF-CA3 synapse require the activation of *β*-adrenergic receptors [47]. In addition, ECT treatment can increase dopamine regulation in MF [48]. The antidepressant drugs can enhance D1 receptor-dependent synaptic potentiation in the MF-CA3 [49]. Vagus nerve stimulation (a treatment for depression) can cause an increase in PP-CA3 synaptic transmission [50]. In an animal model of depression induced by chronic unpredictable mild stress (CUMS), LTP, basal synaptic transmission, and dendrite spine density were decreased in the CA3-CA1 synapse [51]. The CA3-CA1 synapse is also thought to be involved in the acquisition of associative learning [52]. CA3-CA3 synapses are widely distributed [53] and have strong plasticity [54]. Studies have shown that the spike timing-dependent plasticity of the CA3-CA3 synapses contributes to information storage and retrieval [55].

#### 2.2.2. Dentate Gyrus

Apoptosis in the CA1 and CA4 regions and the DG can be observed in patients with MDD [56]. Patients with ECT treatment that had larger right CA4/DG volumes were associated with symptom remission [57]. DG plasticity changes are also involved in the mechanism of some antidepressant drugs. Tianeptine is a special tricyclic antidepressant. It mainly acts on the 5-HT system, which can increase the activity of hippocampal pyramidal cells and the reuptake of 5-HT by hippocampal neurons (as opposed to traditional antidepressants) [58]. Tianeptine was reported to reduce DG apoptosis in a tree shrew model of depression [59]. Maternal separation (MS) can impair learning and memory and cause depression-like behavior. Studies have shown that MS can induce the apoptosis in the DG and reduce cell proliferation in rats [60]. The expression of glutamate receptor 1 and protein kinase B phosphorylation in the DG were also decreased [60]. These factors may be involved in depression caused by MS. Fluoxetine, a selective 5-HT reuptake inhibitor widely used in clinical practice, can selectively inhibit 5-HT transporter and block the reuptake of 5-HT by the presynaptic membrane, thereby producing antidepressant effects. A study has shown that after 2 weeks of MS in rats, 7 days of fluoxetine treatment can reverse cell apoptosis, increase cell proliferation (reduce the number of terminal deoxynucleotidyl transferase-mediated dUTP gap terminal marker-positive cells), and has an antidepressant effect [61]. Chronic stress can damage LTP in the DG of rats, but this effect is reversible [62].

The effect of sirtuin (SIRT) 2 knockdown in the DG is similar to that of chronic stress, and both downregulate plasticity-related genes [63]. In addition, chronic stress reduces SIRT1 activity in mice, leading to depression-like behavior [64]. Physical activity can modulate LTP and LTD in DG of rodents, but the effects are complex and depend on the studies and the conditions (exercise regime, duration, and intensity) [65]. Both acute stress and dexamethasone injection increased the release of somatostatin (SST) in DG hilar cells [66]. Several studies have shown that SST promotes LTP in the DG [67]. However, the opposite effect has also been reported [68]. The reason for this phenomenon may be that SST has different effects on different hippocampal synapses.

There are other factors, such as dopamine [69], 5-HT [70], sex hormones (estradiol, testosterone) [71], paracrine signaling factor (Wnt [72], Notch1 [73]), proinflammatory cytokines [74], and epigenetic changes [75], that are also involved in regulating DG plasticity, which we will not elaborate here.

### 2.3. Glutamate Receptor Involvement in Synaptic Plasticity

Glutamate receptors are mainly divided into two categories: one is ionic receptors including the N-methyl-d-aspartate receptor (NMDAR), the *α*-amino-3-hydroxy-5-methyl-4-isoxazole propionic acid receptor (AMPAR), and the kainite receptor (KAR), and the metabolic receptor, mGluR. These receptors are closely related to hippocampal plasticity to varying degrees.

#### 2.3.1. N-Methyl-d-aspartate Receptor (NMDAR)

NMDAR has multiple subtypes, including NR1 and NR2A-D. The function of NMDAR is determined by the different functions, structure and distribution of various subunits, and different combinations of subunits [76]. Generally, NR1 is considered to be an important component of NMDAR functionality [77]. NMDAR ligand-gated ion channels are regulated by glycine [78], and glycine has a binding site on NR1 [79]. Previous studies have shown that NMDAR is mainly distributed in the postsynaptic density (PSD) of the postsynaptic membrane. Studies in recent years have confirmed that NMDAR is also distributed in the presynaptic membrane and non-PSD areas [80]. PSD95 participates in NMDA receptor regulation [81]. PSD95 expression promotes the maturation of excitatory (glutamatergic) synapses, increasing the number and size of dendritic spines [82]. PSD95 stabilizes NMDAR by binding to GluN2B or degrading STEP61 [83]. The activity-dependent regulation of STEP61 and its substrates GluN2B and GluA2 (a subunit of AMPAR) may contribute to the homeostasis of excitatory synapses [84]. In the absence of stimulation, the NMDA ion channel is not opened due to the blocking of Mg2+. Under various stimuli, the postsynaptic membrane depolarizes. The blocking effect of Mg2+ disappears, and Ca2+ and Na+ enter the cell, increasing free Ca2+ in the cell and producing various biochemical reactions. NMDAR-dependent LTP is widely reported in the hippocampus [85]. Ketamine is an NMDAR antagonist, and its rapid antidepressant mechanism has been confirmed in human and animal models. Ketamine can reverse the decrease of hippocampal CA3 and DG dendritic spine density in depressed mice, activate AMPAR, and increase BDNF expression and release and activation of the mammalian target of rapamycin (mTOR) [86]. Depressed rats treated with ketamine exhibit increased LTP and levels of EPSCs mediated by NMDA receptors in the hippocampus [87]. In addition to PSD95, NMDAR also binds with multiple molecules including CaMKII [88] and microtubule-associated protein 2 [89], to form a multiprotein complex, which plays a role in plasticity, learning, and memory.

#### 2.3.2. *α*-Amino-3-hydroxy-5-methyl-4-isoxazole Propionic Acid Receptor (AMPAR)

AMPAR regulates synaptic plasticity through changes in the number of postsynaptic membranes (insertion or removal of AMPAR). AMPAR regulates synaptic transport during LTP through two ways: lateral movement and exocytosis [90]. Lateral diffusion exchanges AMPAR at the dendritic spines, depending on the spinal morphology [91]. AMPAR surface diffusion is the key to hippocampal LTP and learning [92]. Acute stress can increase the AMPAR phosphorylation and surface expression in the hippocampal CA1 region of mice and alleviate LTP impairment [93]. Another way to recruit AMPAR to the synapse is through exocytosis [94]. Cyclin Y inhibits AMPAR exocytosis of dendritic spines, thereby inhibiting LTP [95]. This exocytosis in LTP passes through the RAS/ERK signaling pathway [96]. In addition, the silent synapses (the synapse only expresses NMDAR before inducing LTP) express AMPAR after inducing LTP and then, they become a functional synapse [97]. Several studies have suggested that hippocampal LTD is associated with AMPAR endocytosis. Calcyon can regulate endocytosis, and knocking out the gene that encodes calcyon leads to the LTD disappearance in the hippocampal CA1 region [98]. Inhibition of AMPAR endocytosis can increase the AMPAR level and decrease LTD [99]. However, one study suggested that AMPAR-induced LTD was the result of inhibiting exocytosis rather than enhancing endocytosis [100].

#### 2.3.3. Kainate Receptor (KAR)

There has been less focus on KAR than on the other two ionic glutamate receptors. However, the function of KAR associated with plasticities such as the modulation of excitability [101], transmitter release [102], neuronal development [103], and neurogenesis [104]. The neto protein is an important auxiliary protein that regulates the KAR in many interneurons [105]. The distribution of KAR also shows some differences, and it is expressed in the presynaptic regions, in addition to the postsynaptic regions [106]. Furthermore, the bidirectional regulation of KAR is also considered to play an important role in plasticity [107]. Several studies have found a link between KAR subtypes and depression. GluR7 is a susceptibility gene associated with recurrent MDD [108]. GluK4 (a KAR subunit) deficiency is associated with increased cognitive ability [109]. Rodent models have also confirmed that GRIK4 (GluK4 gene) knockout causes an antidepressant phenotype [110].

Chronic stress and corticosteroids increase KAR subunit mRNA expression in rats [111]. KAR is reported to be involved in glutamate release in the CA1 and CA3 regions [112]. The short-term and long-term potentiation of KAR participation in the hippocampal MF synapse has been generally confirmed, in which presynaptic KAR plays a central role [113]. Similarly, KAR is also involved in the induction and expression of hippocampal LTD [114]. KAR regulates not only the glutamatergic system but also the GABAergic system. KAR inhibits GABA release and the synaptic transmission to CA1 [115]. However, another study came to the opposite conclusion. KAR increased the efficacy of GABAergic synapses [116]. This effect could be related to the agonist concentration, but the specific mechanism is still unclear.

#### 2.3.4. Metabolic Receptor (mGluR)

mGluR is a type of G-protein-coupled receptor and is divided into three categories (I, II, and III) with 8 subtypes (mGluR1-8). Type I mGluRs include two subtypes: mGluR1 and mGluR5. Chronic stress can increase type I mGluR-mediated LTD in the hippocampal CA1 region [117], while acute stress has only a promoting effect [118]. This phenomenon indicates that type I mGluR-mediated plasticity changes require repeated stress stimulation. Besides, DHPG (a type I glutamate receptor agonist) induced synaptic plasticity of the Schaffer collateral NMDAR (induced LTD) [119]. Studies on type I mGluRs mainly focus on the feedback circuit of the hippocampal stratum oriens, in which type I mGluRs induce LTP [120]. The type I mGluRs induced anti-Hebbian LTP in interneurons of rat hippocampal stratum oriens [121]. Type I mGluRs are also involved in TEA-induced LTP in rat MF-CA3 synapses [122]. Studies have shown that type I mGluR-mediated synaptic plasticity occurs via the *β*-arrestin signaling pathway [123]. The TRPC1 channel plays a critical role in the process of mGluR5-regulated plasticity [124].

Although type II and III mGluRs are rarely reported in terms of hippocampal plasticity, their important role cannot be ignored. Fluoxetine combined with a low-dose ly379268 (a mGluR2/3 agonist) can increase cell proliferation and neurogenesis of cultured cerebellar granule neurotransmitters and shorten the incubation period required for the downregulation of hippocampal *β*-adrenergic receptors [125]. The study of type III mGluRs showed that MS significantly reduced the expression of mGluR4 in the hippocampus of rats, while fluoxetine reversed the changes induced by MS [126]. Behavioral pharmacology studies have shown that LSP4-2022 (a selective agonist of mGluR4) has a strong effect in promoting depression in mice. This effect does not exist in mGluR4-knockout mice, but whether an mGluR4 antagonist can induce an antidepressant effect needs further verification [127]. In mGluR4-knockout mice, improved spatial learning is associated with hippocampal LTP [128]. AMN082 (an mGluR7 allosteric agonist) produces antidepressant-like effects by regulating glutamatergic signaling [129]. mGluR7-knockout mice show antidepressant phenotype, causing HPA axis dysregulation and increasing hippocampal BDNF protein levels [130].

### 2.4. Hippocampal Apoptosis and Neurogenesis

The neurogenesis hypothesis is to some extent an extension of the BDNF hypothesis. Apoptosis of hippocampal neurons has been observed in MDD patients and rodent depression models [131, 132]. Apoptosis of hippocampal neurons may be one of the causes of depression. Venlafaxine is a dual inhibitor of 5-HT and norepinephrine reuptake, which can inhibit the reuptake of NA and 5-HT and slightly inhibit the reuptake of dopamine. Venlafaxine also inhibits hippocampal neuron apoptosis in depression by upregulating the expression of BDNF in the hippocampus of rats [133].

Hippocampal neurogenesis is the process by which adult neural stem cells become progenitor cells and then functional DG cells, providing functional and structural plasticity [134]. Although the number of newborn neurons is much smaller than that in the hippocampal granular cell layer, these cells have important implications in hippocampal function. Increased adult hippocampal neurogenesis can reduce depression-like behavior in mice [135]. Chronic corticosterone injection reduced the neurogenesis of DG in rats [136] and the dendritic complexity of mature granule cells [137]. Adult neurogenesis altered the excitability of the DG [138] and enforced chronic stress adaptability by inhibiting mature granulosa cells in the ventral DG [139]. Ketamine increased DG cell proliferation in depressed rats [140]. Stress and corticosterone also inhibit progenitor cell proliferation, possibly by increasing nitric oxide levels [141].

### 2.5. Glial Cell Plasticity

Glial cells are another prominent cell type in nerve tissue and are widely distributed in the central and peripheral nervous systems. The glial cells in the central nervous system mainly include astrocyte, oligodendrocyte, and microglia. There is evidence that astrocytes affect adult neurogenesis in hippocampal DG [142]. The activity of p38*α* mitogen-activated protein kinase (MAPK) in astrocytes is required for hippocampal LTD [143]. Chronic mild stress-induced depression may be related to a decrease in the number of astrocytes and the activation of microglia [144]. The pruning of synapses by microglia is necessary for brain development [145]. Microglia reshape synapses through presynaptic phagocytosis and spine head filopodia induction. Microglial inhibitors can eliminate the decrease in hippocampal LTP and LTD caused by peripheral inflammation [146]. In CUS-induced depression model rats, the number of oligodendrocytes in the hippocampal CA3 and DG regions is decreased [147]. Oligodendrocyte depolarization can induce short-term and long-term plasticity in hippocampal white matter [148]. Oligodendrocytes can regulate axonal excitability and nerve conduction [149]. The inhibition of oligodendrocyte formation impairs memory consolidation in mice [150]. Myelin basic protein is a marker of mature oligodendrocytes. Chronic social frustration stress can cause changes in the plasticity of ventral hippocampal myelin, but this effect depends on genetic background [151].

## 3. Role of Brain-Derived Neurotrophic Factor in Depression

**Figure 1:**
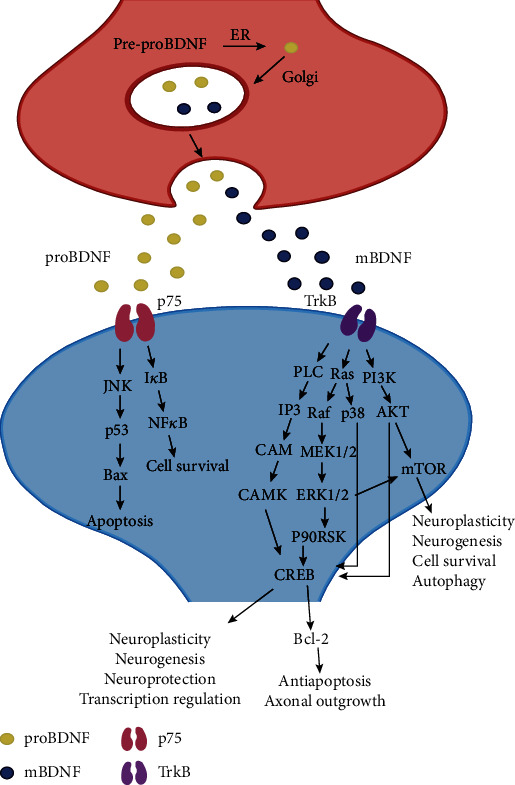
Diagrams of the BDNF-relevant signaling pathways. BDNF: brain-derived neurotrophic factor; ER: endoplasmic reticulum; TrkB: tropomyosin receptor kinase B; PLC: phospholipase C; IP3: inositol triphosphate; CAM: calmodulin; CAMK: CAM-dependent protein kinase; p90RSK: 90 kDa ribosomal S6 kinase; ERK1/2: extracellular-regulated kinase 1/2; PI3K: phosphoinositide 3-kinase; AKT: protein kinase B; mTOR: mammalian target of rapamycin; CREB: cAMP-response element binding protein; JNK: c-Jun N-terminal kinase; Bax: Bcl-2-associated X protein; NF-*κ*B: nuclear factor-*κ*B; I*κ*B: an inhibitor of NF-*κ*B.

The neurotrophic factor and neurogenesis hypotheses connect BDNF with plasticity and depression. Clinical studies have shown that plasma BDNF levels are reduced in patients with depression [152], and antidepressant treatment can increase BDNF levels [153]. BDNF is an indispensable factor in the antidepressant effects of ketamine [154]. BDNF plays an important role in regulating hippocampal plasticity. BDNF regulates LTP in the hippocampus by enhancing synaptic responses [155]. In addition, BDNF also affects the expression of monoamine genes in the hippocampus [156]. Current studies have mainly focused on BDNF and its precursor (proBDNF) and mature forms (mBDNF) [157]. CUMS caused a decrease in the BDNF/proBDNF ratio in the rodent's hippocampus: BDNF rescued CUMS-induced behaviors and spine loss, while proBDNF resulted in a decrease in spine density [158]. The high-affinity receptor of BDNF is tropomyosin receptor kinase B (TrkB), which has low affinity for p75 [159]. Furthermore, BDNF activates multiple signaling pathways (Figure 1). Therefore, in this section, we focus on these signaling pathways and discuss the role of the BDNF signaling pathway in hippocampal plasticity in depression and antidepressant drugs.

### 3.1. Mitogen-Activated Protein Kinase (MAPK) Pathway

After binding to TrkB, BDNF phosphorylates Shc and alters the conformation of the TrkB-Shc complex [160]. Phosphorylated Shc then acts on the downstream growth factor receptor-bound protein 2 (GRB2) and the son of sevenless (SOS), which in turn activate the MAPK signaling pathway, leading to ERK1/2 phosphorylation [161]. BDNF may increase nuclear CREB activity through 2 main pathways: p38 phosphorylation and 90 kDa ribosomal S6 kinase (p90RSK) phosphorylation [162, 163]. CREB is an important hub in neuroplasticity and neuroprotection [164]. Many examples of antidepressants work through the BDNF/MAPK pathway. For example, the plant *Centella asiatica* supposed to have antidepressant properties improves the memory of chronic electrical stress rats (increased hippocampal BDNF levels) via the BDNF/TrkB/ERK pathway [165]. The plant *Angelica sinensis* plays a neuroprotective effect through the BDNF/CREB/p90RSK signaling pathway [162]. Imipramine, a selective inhibitor of monoamine reuptake, can exert neuroprotective effects via the BDNF/MAPK/Bcl-2 pathway [166].

### 3.2. Phospholipase C*γ* (PLC*γ*)

Activated PLC hydrolyzes the membrane component phosphatidylinositol 4,5-bisphosphate (PIP2) to produce a second messenger inositol triphosphate (IP3). IP3 promotes the release of Ca2+ from the cellular calcium reservoir and increases the concentration of Ca2+ in the cytoplasm. Ca2+ then binds to calmodulin (CAM) to transmit the signal. The signal transduction molecule downstream of the Ca2+/M complex is a protein kinase that can be activated by the Ca2+/CAM complex called CAM-dependent protein kinase [167]. One study showed that the TrkB/PLC pathway mediated mouse hippocampal LTP and regulated CREB activity [168].

### 3.3. Phosphoinositide 3-Kinase (PI3K) Pathway

Activated PI3K can catalyze the production of phosphatidylinositol 3,4,5-triphosphate (PIP3). PIP3 activates AKT by binding to its pleckstrin homology (PH) domain and can also activate AKT by activating 3-phosphoinositide-dependent protein kinase 1 (PDK1) [169]. BDNF has been reported to inhibit autophagy through the PI3K-Akt signaling pathway [170].

Most studies on downstream molecules of the BDNF-mediated PI3K-Akt signaling pathway have focused on mammalian target of rapamycin (mTOR). The BDNF/mTOR signaling pathway is involved in the rapid antidepressant mechanism of the antidepressant drug ketamine [154], and ketamine enhances the structural plasticity of dopaminergic neurons through the AMPA receptor-driven BDNF/mTOR signaling pathway [171]. S 47445 (positive allosteric modulator of AMPAR) reduced the motor activity of olfactory bulb-excised mice, showing an antidepressant effect, and reversed the changes in the expression of BDNF/mTOR [172]. Hypidone hydrochloride (YL-0919) showed a faster antidepressant effect than fluoxetine and also reversed the activity of BDNF/mTOR signaling and some key synaptic proteins [173]. Sulforaphane can play an antidepressant effect in chronic mild stress mice and can block the elevation of corticosterone, corticotropin, IL-6, and TNF-*α* in serum of mice [174]. The antidepressant effect of sulforaphane may be through inhibition of the HPA axis and inflammatory response. Furthermore, sulforaphane can reverse the decrease of BDNF and dendritic spine density in depressed mice [175]. Knockdown of hyperpolarization-activated cyclic nucleotide-gated channel 1 (HCN1) increased the excitability of cells in the mouse CA1 region, resulting in showing an antidepressant phenotype [176]. Inhibition of HCN1 can reduce depression and improve learning ability in rats [177]. These effects are all related to the upregulation of the BDNF/mTOR signaling pathway and synaptic transmission. Studies on the binding of mTOR with different proteins to form functional polymer complexes suggested that BDNF/mTOR1 can be used as a research focus for a new generation of antidepressant drugs [178]. The BDNF/mTOR1 signaling pathway may be the target of the antidepressant effect of traditional prescriptions (lily bulb and Rehmannia decoction) [179].

## 4. Caloric Restriction as a Putative Treatment in Depression

**Figure 2:**
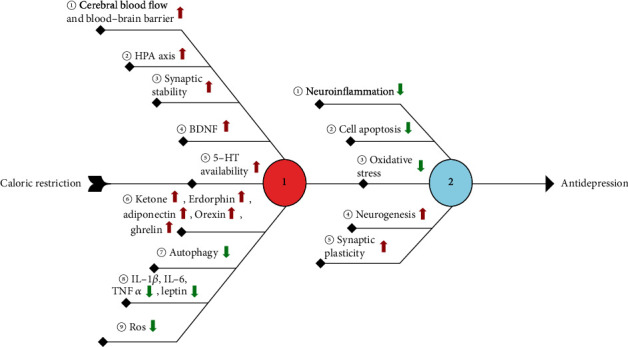
The molecular mechanism involved in the antidepressant effect of caloric restriction.

The antidepressant effect of CR has been confirmed in rodent depression models (Figure 2). During aging, neuroinflammation and oxidative stress increase in the hippocampus, and synaptic plasticity and neurogenesis decrease, but these adverse reactions can be alleviated by CR [180]. CR reduces cell death in the hippocampal CA3 area after kainate administration [181]. At the genetic level, CR reduces basic DNA loss [182] and affects the expression of genes associated with synaptic plasticity in the hippocampal CA1 and CA3 region [183]. CR can stabilize the gene expression of presynaptic proteins [184] and prevent the reduction in key synaptic proteins in the CA3 [185]. In terms of neuroendocrine, CR increased the expression of adiponectin in rodent adipose tissue and blood adiponectin levels [186]. Patients with BD often have decreased plasma adiponectin levels [187]. Adiponectin and antidepressant drugs can improve the symptoms of depression [188]. Adiponectin has a beneficial effect in fighting neuroinflammation [189] and also plays an important role in the remodeling and neurogenesis of dendrites and dendritic spines of mouse hippocampal neurons [190]. All of these factors have beneficial effects on depression. Furthermore, CR can also lead to reduced leptin levels [191]. There have been different research results on the relationship between leptin and depression. Studies have shown that patients with depression have reduced leptin levels [192], and the same conclusions have been reached in animal models of depression [193]. However, in another study, it was found that patients with MDD had increased leptin levels [194]. Leptin is considered to be a proinflammatory factor, and the reduced level caused by CR may have a positive effect on depression [195]. CR also increases hippocampal synaptic plasticity by changing the morphology and function of astrocytes [196]. A study has confirmed that CR can preserve LTP that is lost in the hippocampus of aging rats [197]. cAMP and its response element-binding protein CREB are involved in the CR-mediated regulation of plasticity [198]. However, the regulation of plasticity by CR is bidirectional, as long-term CR decreased hippocampal neurogenesis and granulosa cell density [199] and leads to reduced levels of some lipids in the DG, impairing spatial memory [200], whereas short-term CR has beneficial effects on hippocampal plasticity. Proteomics analysis showed that CR could improve glutamate disorders, impaired protein synthesis, and mitochondrial dysfunction [201].

Depression may be related to impaired hippocampal mitochondrial plasticity, which is restored by antidepressant treatment [202]. Mitochondria contribute to increasing dendritic spines, synapse regeneration, and synaptic plasticity [203]. When mitochondria increase synaptic plasticity, BDNF levels increase [204]. Chronic stress leads to mitochondrial dysfunction and mitochondrial protein imbalance [205]. Importantly, LTP requires a rapid burst and fission of dendritic mitochondria [206]. In terms of treatment, physical exercise can improve posttraumatic stress disorder through an increase in hippocampal mitochondrial function [207]. Mitochondria are associated with BDNF-mediated synapses and vascular plasticity in ECT [208].

## 5. Conclusion

**Figure 3:**
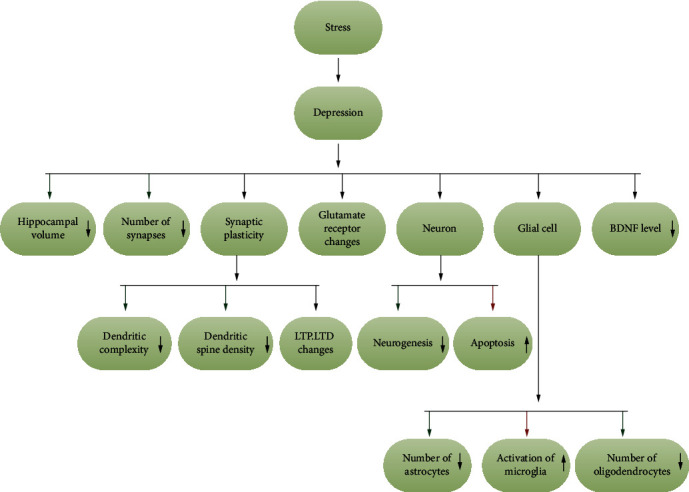
Changes in hippocampal plasticity in depression.

Different degrees of hippocampal plasticity changes have been observed in clinical depression patients and rodent depression models (Figure 3). BDNF plays a central role in hippocampal neuroplasticity, and a variety of changes involve the activation or inhibition of the BDNF signaling pathways. The downstream mTOR signaling pathway has received widespread attention since it was discovered to have a role in the antidepressant mechanism of ketamine. BDNF also promotes neurogenesis and remodeling of synaptic morphology and structure through the activation of the mTOR signaling pathway. However, due to the many side effects of ketamine and the fact that the direct activation or inhibition of the mTOR signaling pathway may have adverse effects on humans, the research on mTOR has been restrained to a certain extent. However, this does not affect its potential as a new generation, fast-acting antidepressant treatment target. The positive effects of CR on a variety of neurological diseases (including depression) have been confirmed. Because CR has almost no side effects and is simple to perform, it does not burden patients further with discomfort or treatment costs. CR may be used as a physical form of clinical antidepressant treatment in the future and support other antidepressant treatments to further reduce the suffering of patients with depression. However, the deeper molecular and cellular mechanisms of CR need to be further explored, and its possible defects should be clarified.
